# Chloroform the Remedy for the Albuminuria of Pregnancy

**Published:** 1890-08

**Authors:** A. W. Griggs

**Affiliations:** Professor Emeritus of the Principles and Practice of Medicine in the Atlanta Medical College, and President of the Medical Association of Georgia


Southern Medical Record.

A MONTHLY JOURNAL OF MEDICINE AND SURGERY.

VOL. XX. ATLANTA, GA., AUGUST, 1890. NO. 8.

### Original Articles.

### CHLOROFORM THE REMEDY FOR THE ALBUMINURIA OF PREGNANCY.

BY A. W. GRIGGS, M. D., PROFESSOR EMERITUS OF THE PRINCIPLES AND PRACTICE OF MEDICINE IN THE ATLANTA MEDICAL COLLEGE, AND PRESIDENT OF THE MEDICAL ASSOCIATION OF GEORGIA.

Tyler Smith, I believe, first pointed out the relation between albuminuria and puerperal eclampsia. This relation exists in the great majority of such cases. It is true that the convulsions may, and do, occasionally occur in women of the nervous temperament, from morbid reflexes, such as happen from pressure of the fetal head upon perineum or the presence of a clot in utero.

Now since albumen is present in the urine in a large percentage of the cases of eclampsia, and since the cure of the albuminuria gives all but absolute immunity against eclampsia, it behooves us to keep on the alert, and if possible, avert the coming storm. This can be done without difficulty, by the timely administration of *chloroform* per orem.

Certainly a more important subject cannot engage the at tention of the profession. It has been urged time and again by myself and others, but it appears that from the meager literature on the subject it has not reached the medical masses, or has been ignored by them through indifference or want of confidence. I now write to induce all to pay heed to the fact, that puerperal eclampsia is easily prevented, without danger to mother or child from the treatment. We especially ask the young medical men to try the method, as we can give positive assurance of its great success. It is the duty of the family physician to make inquiry into the condition of every pregnant woman in the circle of his patrons and give such advice or institute such treatment as will give security against the dangers which may lie in wait.

It is our custom to ask the patient these questions: How far advanced in pregnancy are you? Do the hands and face feel swollen and tight? Are the rings growing too small for the fingers? Do you feel that you are generally swollen over the whole body? Do you suffer from pain over the crown of the head? Do you have any perversion of vision? Do you have any trouble in your speech? Is there any diminution in the amount of urine secreted?

If these questions or only a few of them are answered affirmatively, I am sufficiently satisfied to test the urine, for the convincing proof of the existence of albuminuria, and I am all but sure to find it.

It does not matter in what stage of utero-gestation it may be, eclampsia may be fully expected, unless the morbid processes which are going on be corrected.

If the treatment is delayed, it will not be a fair test. Begin as soon as the case is made out, and if you have a month's time to go on, you will be apt to succeed. Two months will usually be ample time. The plan is to administer chloroform in doses suited to circumstances as to the time you may have for treatment, or as to the urgency of the symptoms.

If we have six or eight weeks we begin with twelve drops and gradually increase the dose up to fifteen or twenty. The nearer the term, the larger the dose should be, though it is generally better to begin with a small quantity and increase it, as may be necessary. These doses are taken before each regular meal time, or three times a day, and also at midnight. The drug is dropped into two tablespoonfuls of water, or more if desired, and the patient directed to hold the breath and swallow promptly, immediately exhaling a portion of air from the lungs, so as to blow off the fumes of the medicine. In very urgent cases, we have given much larger doses with safety. I remember one case to which I was called that was on the very border of eclampsia. She had about a week to go, and was suffering with pain over the crown of the head. She was having one shock after another as if from a powerful battery. Twenty-five drops of chloroform were administered and fifteen were given in a half hour afterward, and the dose was repeated every half hour until she became quiet and composed. The urine, which had been scanty, rapidly increased in quantity, and great relief was afforded. The chloroform was continued in twenty-five drop doses four times daily until she was confined, which ordeal she passed through without an unfavorable symptom. Let me say that we have treated a number of such cases in the last fifteen or twenty years with entire success. Of course they were not all so bad, and we had more time to devote to their management. We not only gave the medicine, but prescribed the adoption of the best hygienic regulations.

During the treatment, the urine should be examined at least once a week, that we may know how to regulate the dose of the chloroform. The treatment should be persevered in as long as there is albumen to be found in the urine. A good rule is to increase the chloroform if the albumen is increasing, and *vice versa*. Do not crowd the patient too freely, but use good judgment and you will be satisfied.

It will be found that the digestion will be improved under the use of the chloroform, and the anasarca will be relieved. We will try to explain in a future article the modus operandi of the great anaesthetic in these cases. It has been claimed that Dr. H. V. M. Miller first instituted this treatment. I do not know when he first used it, nor how he has led to its use. He is one of the most brilliant men in the profession, and worthy to wear its highest honors.

I have tried to get up all that has been written on this subject, but find but little in medical literature. Dr. John G. Westmoreland and myself used this treatment many years ago, but never made any claim of priority. The purpose of this article is to induce gentlemen to adopt the treatment for the sake of science and humanity. That is all.

